# Drug Response-Related DNA Methylation Changes in Schizophrenia, Bipolar Disorder, and Major Depressive Disorder

**DOI:** 10.3389/fnins.2021.674273

**Published:** 2021-05-13

**Authors:** Jiaqi Zhou, Miao Li, Xueying Wang, Yuwen He, Yan Xia, John A. Sweeney, Richard F. Kopp, Chunyu Liu, Chao Chen

**Affiliations:** ^1^Center for Medical Genetics & Hunan Key Laboratory of Medical Genetics, School of Life Sciences, Department of Psychiatry, The Second Xiangya Hospital, Central South University, Changsha, Hunan, China; ^2^Department of Psychiatry, State University of New York Upstate Medical University, Syracuse, NY, United States; ^3^Department of Psychiatry, University of Cincinnati, Cincinnati, OH, United States; ^4^Hunan Key Laboratory of Animal Models for Human Diseases, Central South University, Changsha, Hunan, China

**Keywords:** pharmacoepigenetic, DNA methylation, drug response, schizophrenia, bipolar disorder, major depressive disorder

## Abstract

Pharmacotherapy is the most common treatment for schizophrenia (SCZ), bipolar disorder (BD), and major depressive disorder (MDD). Pharmacogenetic studies have achieved results with limited clinical utility. DNA methylation (DNAm), an epigenetic modification, has been proposed to be involved in both the pathology and drug treatment of these disorders. Emerging data indicates that DNAm could be used as a predictor of drug response for psychiatric disorders. In this study, we performed a systematic review to evaluate the reproducibility of published changes of drug response-related DNAm in SCZ, BD and MDD. A total of 37 publications were included. Since the studies involved patients of different treatment stages, we partitioned them into three groups based on their primary focuses: (1) medication-induced DNAm changes (*n* = 8); (2) the relationship between DNAm and clinical improvement (*n* = 24); and (3) comparison of DNAm status across different medications (*n* = 14). We found that only BDNF was consistent with the DNAm changes detected in four independent studies for MDD. It was positively correlated with clinical improvement in MDD. To develop better predictive DNAm factors for drug response, we also discussed future research strategies, including experimental, analytical procedures and statistical criteria. Our review shows promising possibilities for using BDNF DNAm as a predictor of antidepressant treatment response for MDD, while more pharmacoepigenetic studies are needed for treatments of various diseases. Future research should take advantage of a system-wide analysis with a strict and standard analytical procedure.

## Background and Motivation

Shizophrenia (SCZ), bipolar disorder (BD), and major depressive disorder (MDD) are severe psychiatric disorders, conferring lifelong disability (Hyman, 2012; GBD 2015 Disease and Injury Incidence and Prevalence Collaborators, 2016). A majority of patients with these psychiatric disorders receive medication as the first-line treatment (Moore et al., 2009; Pratt et al., 2017). Therapeutics for SCZ, BD, and MDD are generally based on similar classes of molecules, targeting similar pathways, with distinct doses and proper combinations. However, drug selection is clinically subjective and treatment typically requires weeks of symptom evaluation to determine treatment efficacy. A large proportion of patients fail to respond to first-line drug treatment. For example, the Sequenced Treatment Alternatives to Relieve Depression trial (STAR^∗^D) study reported that only 35% of patients remit after their primary antidepressant treatment (Rush et al., 2006; Katz et al., 2012). This illustrates the importance of developing biomarkers that can support decisions regarding optimal drug choice, and identify likely poor responders as quickly as possible. This emphasizes the need for a better biomarker-based stratification of patients that could facilitate treatment planning.

Pharmacogenetic approaches for guiding the treatment of psychiatric disorders has been a rapidly expanding area of research in the last two decades (Nelson et al., 2016). The main hypothesis of these studies was that genetic variants could predict the influences of drug treatment. Numerous pharmacogenetic studies have investigated the genetic contribution to the treatment response of these disorders (Baum et al., 2008; Brandl et al., 2016; Shi et al., 2017; Yu et al., 2018). Clinical practice for pharmacogenetic findings is emerging, as a few of the Food and Drug Administration (FDA) approved drug labels changed for SCZ and MDD. Although psychiatric disorders are thought to be highly heritable, gene-environment interactions are relevant, and results of pharmacogenetic studies have been inconsistent, limiting the clinical utilization of this approach.

Emerging evidence suggests that epigenetic marks could be used as a predictor of drug response for psychiatric disorders (Chan and Baylin, 2012; Heerboth et al., 2014). Dysregulation of epigenetic events can be pathological, and associated with SCZ, BD, and MDD (Mill et al., 2008; Sabunciyan et al., 2012; Guintivano et al., 2013; Chen et al., 2014; Liu et al., 2018). This implies that biological pathways and cellular processes are under the impact of epigenome status. Unlike genetics, epigenetic status is dynamic and could better reflect various environmental events during disease progress and drug treatment. Because of the reversibility of epigenetic events, we postulate that modulation of epigenetic regulators could be valuable for therapeutic potential. Pharmacoepigenetics may be more promising for guiding the treatment of psychiatric disorders than pharmacogenetics.

The recent advent of interest in pharmacoepigenetics of psychiatric disorders is an alternative research avenue. The epigenetic modification known as DNA methylation (DNAm), one of major epigenetic modifications, is the product of the interaction between genetic variants and environmental influence, and for this reason might be a better predictor of treatment outcomes (Kubota et al., 2012; Moore et al., 2013). Measurable in peripheral blood, alterations of DNAm have been reported to be involved in both the pathology and drug treatment of SCZ, BD, and MDD (Schroeder et al., 2012; Vialou et al., 2013; Jaffe et al., 2016). Drugs may exert their effects by reversing these DNAm deregulations. Moreover, some FDA-approved drugs, like clozapine and sulpiride, have demonstrated the ability to activate brain DNA demethylation (Dong et al., 2008). Therefore, DNAm is a promising molecular approach to study mechanisms and prediction of drug response in psychiatric disorders (Goud Alladi et al., 2018; Lisoway et al., 2018). However, the reproducibility of those results, crucial for their clinical application, has not been thoroughly evaluated.

**FIGURE 1:**
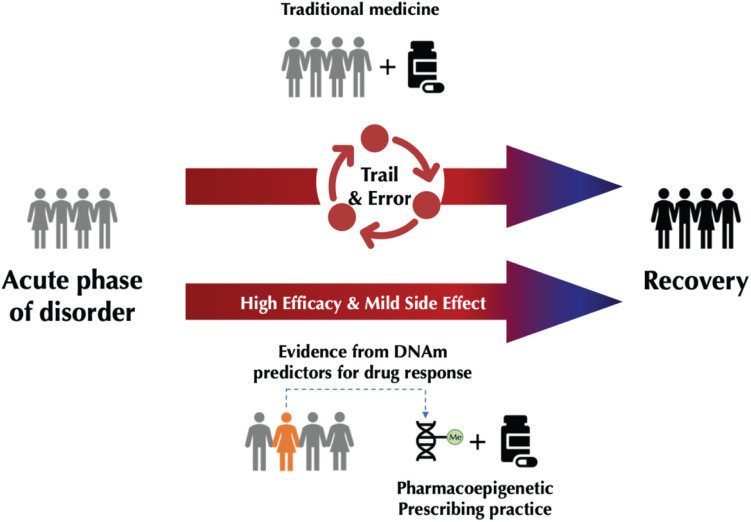
Schematic representation of traditional medicines vs. personalized medicine using pharmacoepigenetic approaches.

We systematically reviewed the literature on DNAm and drug response in SCZ, MDD, and BD to determine if epigenetic markers have produced any reproducible finding as predictors of drug response. In this review, we focused on studies providing information about the effect of medications on DNAm and the utility of DNAm as a predictor of drug treatment for major psychiatric disorders (Figure 1). First, we evaluated the reproducibility of reported changes of DNAm-related genes associated with drug response in each disorder. We also evaluated whether the results of candidate gene studies could be reproduced in genome-wide studies. Finally, we discuss limitations of existing studies and provide recommendations for future research.

## Methods

We systematically reviewed published studies that examined DNAm in relation to drug response in SCZ, BD, or MDD, using the PubMed, Web of Science, and MEDLINE with the following search builder: (((((((pharmacogenetic) OR pharmacogenomic) OR pharmacoepigenetic) OR drug response) OR treatment) OR drug efficiency) OR #DRUG#) AND ((((DNA methylation) OR methylation) OR epigenetic) OR EWAS) AND (#DISORDER#). #DRUG# is the major drug for each disorder: antipsychotic, mood stabilizer, lithium, antidepressant, selective serotonin reuptake inhibitor (SSRI) and selective norepinephrine reuptake inhibitor (SNRI). #DISORDER# is one of the major psychiatric disorders: SCZ, BD and MDD. The inclusion criteria were the following: (1) published in English before April 2020; (2) assessed drug response on DNAm levels; (3) sample size larger than 10. Studies on animal and cell culture models were excluded. We discarded one study testing for drug-related co-methylation modules for BD, since it did not directly compare the efficacy of the individual drugs and was incompatible with the other studies. The literature selection followed the standard by Preferred Reporting Items for Systematic Reviews and Meta-Analyses (PRISMA) (Liberati et al., 2009).

**FIGURE 2:**
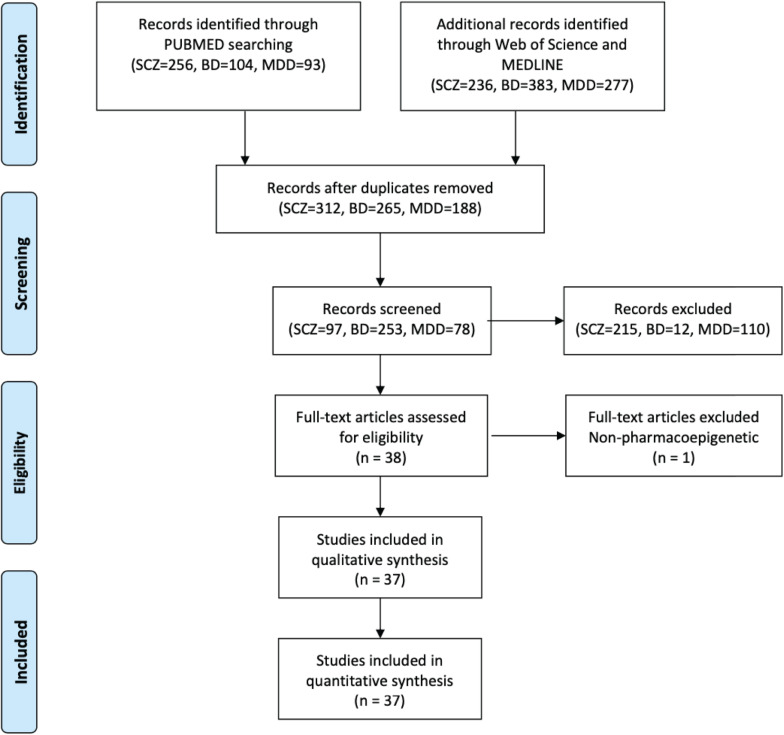
Flowchart of data selection using the Preferred Reporting Items for Systematic Reviews and Meta-Analyses (PRISMA).

**FIGURE 3:**
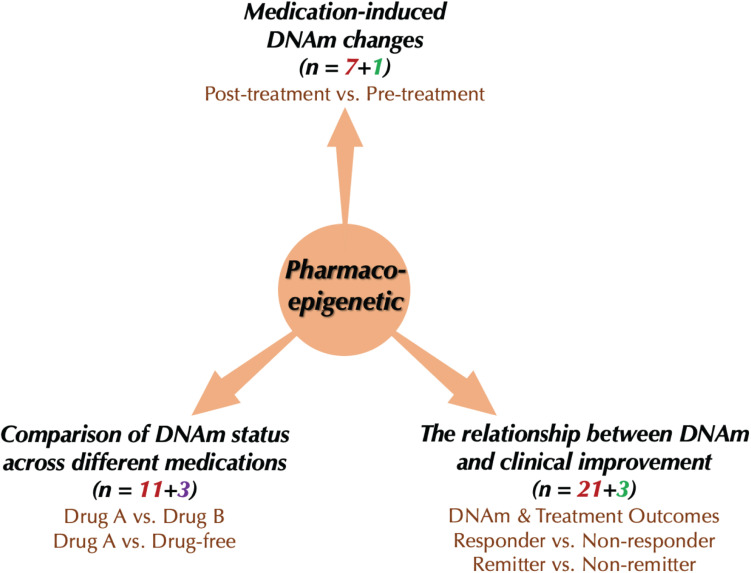
Current research types of pharmacoepigenetics in psychiatric disorders. The red number represents the number of “candidate gene studies,” the green color means the number of “genome-wide studies,” and the purple one means the number of “global methylation studies.”

We included 37 publications about drug response-related DNAm in SCZ, BD, and MDD (Figure 2). Studies included SCZ (number of studies, *n* = 7), BD (*n* = 6), MDD (*n* = 18), and cross-disorder studies (BD and MDD, *n* = 2; SCZ and BD, *n* = 4). Since the studies involved patients of different treatment stages, we partitioned them into three groups based on their primary focuses (Figure 3): (1) medication-induced DNAm changes; (2) the relationship between DNAm and clinical improvement; and (3) comparison of DNAm status across different medications. To evaluate the reproducibility of published changes of DNAm-related genes associated with treatment response in psychiatric disorders, we examined the stability of the reported changes in each disorder. Here, we defined stability as the presence of significant results (*p* < 0.05) that were consistent in more than one independent study. We also evaluated whether the results of candidate gene studies could be reproduced in genome-wide studies.

## Key Concepts, Definitions, and Principles in Pharmacoepigenetics

Pharmacoepigenetics research in psychiatry focuses on the effects of treatments on DNAm and their potential influences on treatment response. The main aim of pharmacoepigenetics is the identification of DNAm of specific genes that are associated with treatment outcomes of psychiatric disorders, with the ultimate goal of translating this information into strategies that can support personalized medicine.

### DNA Methylation

DNA methylation is the epigenetic modification by which methyl groups are added to DNA nucleotides, primarily cytosine, and adenine. DNAm is thought to have a prominent influence on the structure and functions of DNA, particularly in the regulation of gene expression. However, DNAm regulates gene expression differently according to its genomic context. Previous studies revealed that DNAm levels of CpG sites near the transcription start sites could repress gene expression levels, while DNAm levels of gene body CpGs could activate the expression of their target genes (Holliday and Pugh, 1975; Hellman and Chess, 2007; Jones, 2012; Moore et al., 2013). Conflicting results were reported regarding intergenic CpGs: some claimed that they might regulate gene expression less frequently (Chen et al., 2014; Wagner et al., 2014; Liu et al., 2018), while others suggested that intergenic CpGs could repress the gene expression (Gaudet et al., 2004; Hutnick et al., 2010; Moore et al., 2013). Though CpGs are the primary sites for DNAm, it has also been observed at non-CpG sites (CpA, CpT, and CpC) in mammals (Pinney, 2014). Non-CpG methylation sites are common in several tissue types, including skeletal muscle (Yan et al., 2011) and brain (Lister et al., 2013; Guo et al., 2014). However, the functions of non-CpG methylation are still largely unknown and information on their function is just beginning to emerge. Our review includes only studies on the DNAm at CpG sites.

Tissues from postmortem samples, preserved clinical specimens from humans, can be used to study DNAm. For study pharmacoepigenetics for psychiatric disorders, peripheral tissues, such as blood, saliva, and buccal cells, are useful for developing clinically informative biomarkers. Psychiatric disorders are considered primarily a brain dysregulation. Therefore, we prefer to investigate genomic or epigenomic features in peripheral tissues that are similar to brain. Given that DNAm signals can be tissue-specific, it is important to establish the pros and cons of different types of tissue for investigation (Mill and Petronis, 2007).

Several studies investigated correlations of DNAm in blood and multiple regions of the brain, with inconsistent results (Davies et al., 2012; Horvath et al., 2012; Kaminsky et al., 2012; Masliah et al., 2013; Walton et al., 2016). Studies also indicated that DNAm in saliva and buccal cells were more informative surrogate tissues than blood (Lowe et al., 2013; Smith et al., 2015). To address this issue, Braun et al. (2019) performed genome-wide DNAm comparison across the human brain, blood, saliva and buccal cells. They proposed that to determine the optimal surrogate tissue for representing brain DNAm, the DNAm patterns specific to the genomic region of interest between these two tissues must be considered (Braun et al., 2019). More importantly, the proxy tissue should have similar responsive changes to environmental influences such as drugs. Building a reliable peripheral-brain relationship will be critical in proving the biological relevance and illustrating the underlying biological mechanisms of available *in vivo* human tissue for clinical application.

Laboratory techniques have been developed for measuring DNAm not only at global methylation levels but also individual nucleotide levels. In this review, the research strategies used to assess drug response-related DNAm included target analyses for candidate genes, the discovery of genome-wide DNAm with microarrays, and assessment for global methylation. Microarray-based technologies such as Illumina Infinium HumanMethylation27 BeadChip^®^ Array (HM27K) (Bibikova et al., 2009), Infinium^®^ HumanMethylation450 BeadChips (HM450K) (Dedeurwaerder et al., 2011, 2014) and Infinium^®^ MethylationEPIC BeadChip (EPIC) (Moran et al., 2016), have been widely used for methylation profiling. Methylated DNA immunoprecipitation sequencing (MeDIP), Reduced Representation Bisulfite Sequencing (RRBS) and others are also used for DNAm measurement (Song et al., 2005; Couldrey and Cave, 2014). Whole-genome bisulfite sequencing is the gold standard for measuring CpG and non-CpG methylation, but due to its cost, few published pharmacoepigenetic studies have used it.

### Research Strategies

The most widely used approach for studying drug response-related DNAm is still a “candidate gene” method. We found 31 studies of 26 candidate genes completed in the three disorders of interest to us, and findings from 19 of them (73%) reached nominal significance (*p* < 0.05). These candidate gene studies only focus on a limited number of genes, based on their established association with a disorders’ pathogenesis or hypothesized relation to response to drug treatment. Although previous candidate gene studies have identified target genes that may predict or reflect the clinical effect of drug response, most studies had limited statistical power, a concern especially for dense array data. Because of this limitation, the candidate gene method can provide little comprehensive understanding regarding the mechanism of action of drugs, but can only confirm previous knowledge or test relevance of a particular gene methylation (Harrison, 2015).

Along with next-generation sequencing, array-based analyses are commonly used in DNAm measurement to assess site-specific methylation in the genome. These genome-wide analyses offer a non-biased experimental approach to identify novel candidates (Kurdyukov and Bullock, 2016), but we found only 3 studies that that implemented genome-wide analysis, using microarrays to evaluate the drug response. These studies identified several loci associated with clinical improvement, almost all of which were not previously predicted to be relevant. Unfortunately, genome-wide DNAm measurement faces two challenges, first its cost (added to costs of a clinical trial) and second, the challenge of having large samples in a study of patients receiving controlled treatment regimens. These problems limit the size of data sets, and therefore statistical power, for array analysis of drug-related treatment effects. However, genome-wide approaches using a microarray do provide more comprehensive data and could identify novel and unexpected DNAm effects (Barros-Silva et al., 2018).

Additionally, three studies evaluated global DNAm in a specific tissue for a cross-medications comparison. Global DNAm reflects the DNAm status of total genomic content within a sample. Several methods exist to assess global DNAm status, including enzyme-linked immunosorbent assays (ELISA), the use of a previously validated protocol of Linear Interspersed Nuclear Element 1 (LINE- 1) and the use of restriction enzymes (Burghardt et al., 2020). However, this approach only assesses DNAm changes at the genome level, and thus does not further provide data on region-specific DNAm changes to characterize critical regulatory regions (e.g., CpG islands and promoter regions).

## Pharmacoepigenetic Findings in SCZ, BD, and MDD

The included pharmacoepigenetic studies were divided into three types: (1) medication-induced DNAm changes; (2) relationships between DNAm and clinical improvement, and (3) comparison of DNAm status across different medications (Figure 3). In this section, we summarize recent findings from pharmacoepigenetic studies in SCZ, BD and MDD to evaluate the reproducibility of drug response-related DNAm changes in these disorders.

### Medication-Induced DNAm Changes

The changes of DNAm after a period of drug treatment were examined in studies comparing DNAm changes between pre- and post-treatment, which could reflect the effects of drug treatment directly. There were eight studies (Table 1, SCZ = 2, BD = 1, and MDD = 5) that examined medication-induced DNAm changes, including seven that were candidate gene studies and one genome-wide study.

**TABLE 1:** summary of studies exploring medication-induced DNAm changes.

Studies	Genome Region analyzed	Sample size	Drugs (duration)	Tissue	Platform	Main results
**Schizophrenia**						
Venugopal et al., 2018	IL6 Promoter regions	40 SCZ	Co-medications (3 months)	Blood	BIMSA	• Increase of DNAm at IL6 promoter region after drug treatment (*p* < 0.001); • No significant differences in DNAm at IL6 promoter region between endpoint and controls (*p* = 0.17)
Kinoshita et al., 2017	Whole genome	21 SCZ	Clozapine (1 year)	Blood	HM450K	• 29134 CpG sites were differentially methylated after treatment without correction for multiple testing (*p* < 0.05)
**Bipolar disorder**						
Zhang et al., 2018	COMT&PPIEL Promoter regions	150 BD; 50 HC	Co-medications (18 months)	Blood	MS-PCR	• No significant differences in DNAm status at COMT and PPIEL promoter region after 12 months treatment compared with controls (*p* < 0.05)
**Major depressive disorder**						
Tadić et al., 2014	BDNF Exon IV promoter region	39 MDD	Monoaminergic drugs (6 weeks)	Leus	NA	• No significant differences in DNAm status at BDNF promoter region between pre- and post-treatment
Wang et al., 2018b	BDNF Promoter regions	44 MDD	Escitalopram* (8 weeks)	Blood	Illumina Hiseq	• Increase of DNAm at BDNF after escitalopram treatment (*p* < 0.01) • REMs: increase of DNAm after escitalopram treatment • NREMs: no significant differences in DNAm after escitalopram treatment
Okada et al., 2014	5-HTT CpG island at the 5′ region	40 MDD	ADs (6 weeks)	Blood	MCS	• Increase of DNAm at CpG 3 of 5-HTT (*p* = 0.0004, Bonferroni set at *p* < 0.0017)
Kahl et al., 2016	GLUT1&GLUT4 Promoter regions	37 MDD	ADs (6 weeks)	Blood	Bisulfite sequencing	• REMs: decrease of DNAm at GLUT1 gene after treatment (*p* < 0.05) • NREMs: increase of DNAm at GLUT1 gene after treatment (*p* < 0.05) • No significant differences in GLUT4 between pre- and post-treatment
Wang et al., 2018a	HTR1A&HTR1B Promoter regions	44 MDD	Escitalopram* (8 weeks)	Blood	Illumina Hiseq	• No significant differences in average DNAm of HTR1A/1B after treatment • Increase of DNAm at 4 CpG sites in HTR1B after treatment (*p* < 0.05)

Bisulfite Sequencing DNA Methylation Analysis, BIMSA; Leukocytes, Leus; MassARRAY Compact System, MCS; Methylation-specific Polymerase Chain Reaction, MS-PCR; Healthy Control, HC; Remitters, REMs; Non-remitters, NREMs; Antidepressants, ADs. *Monotherapy was permitted for co-medications with other psycho-pharmacological agents or medications for insomnia.

For candidate gene studies, there were 9 candidate genes included and only 5 reached nominal significance (*p* < 0.05). Of those five, only brain-derived neurotrophic factor (BDNF) was analyzed for MDD patients in 2 independent studies (Tadić et al., 2014; Wang et al., 2018b). A significant increase of DNAm in BDNF after 8 weeks of escitalopram treatment for 44 MDD patients based on blood samples was reported by Wang and collaborators (Wang et al., 2018b). The other study did not find significant DNAm changes in leukocytes after 6 weeks of monoaminergic drug treatment for 39 MDD patients (Tadić et al., 2014). One explanation for this discrepancy is that escitalopram belongs to the SSRI class which has distinctly different mechanisms of action from monoaminergic drugs. The treatment duration and tissue types were also different between these two studies.

The genome-wide study identified 29,134 sites (*p* < 0.05) that were significantly differentially methylated after 1 year of treatment with clozapine for 21 SCZ patients based on blood samples, using HM450K assays (Kinoshita et al., 2017). This relatively small study has not been replicated to date. We further evaluated whether the results of candidate gene studies for antipsychotics treatment could be reproduced in genome-wide studies. We found none of those significant candidate genes tested reached a nominal significance in genome-wide analysis.

### The Relationship Between DNAm and Clinical Improvement

More studies explored the relationship between DNAm levels at baseline and clinical improvement after a period of drug treatment. The most frequently used method to assess the clinical improvement is still the scale score (e.g., PANSS, HAM-D). Patients are separated into remission, non-remission, response or non-response based on changes of scale scores. Remission, the symptom of virtual absence of disease, is the goal of treatment (Paykel, 1998). Response is defined as a clinically meaningful reduction in symptoms (e.g., a reduction of at least 50% in baseline symptom levels) (Aaronson et al., 2017). In this section, there were 24 studies included (Table 2). Examining the correlation between baseline DNAm and treatment outcomes for patients, these studies address the question of whether pre-treatment DNAm features could predict clinical improvement.

**TABLE 2:** Summary of studies exploring the relationship between DNAm and clinical improvement.

Studies	Genome Region analyzed	Sample size	Drugs (duration)	Tissue	Platform	Main results
**Schizophrenia**						
Tang et al., 2014	HTR1A Promoter region	82 SCZ	Co-medications (10 weeks)	Blood	Bisulfite conversion + PCR	• DNAm at CpG 13 showed a positive correlation with changes in total PANSS scores (*p* = 0.006) and changes in negative factor (*p* < 0.001) • DNAm at CpG 13 was positively correlated with baseline negative factor (*p* = 0.019)
Shi et al., 2017	CYP3A4, CYP2D6, ABCB1, HTR2A, DRD2 Upstream of promoter region	288 SCZ	Risperidone* (4 weeks)	Blood	MassARRAY Analyzer 4	RES vs. NRES: • Decrease of DNAm at CpG_193 (*p* = 0.0008, *q* = 0.012), CpG_242:244:250 (*p* = 0.000051, *q* = 0.00076) and CpG_284 (*p* = 0.0023, *q* = 0.034) in CYP2D6 • Increase of DNAm at CpG_−367:-372:−374 (*p* = 0.0018, *q* = 0.028), CpG_−222(*p* = 0.012, q > 0.05) and CpG_−243 (*p* = 0.02, q > 0.05) in CYP3A4 • Decrease of DNAm at CpG_−36 (*p* = 0.000091, *q* = 0.0014), CpG_−258 (*p* = 0.000082, *q* = 0.0013), CpG_−296 (*p* = 0.000091, *q* = 0.0014) in CYP3A4
Kinoshita et al., 2017	Whole genome	21 SCZ	Clozapine (1 year)	Blood	HM450K	• Increases in DNAm of the CREBBP (cg05151055, *p* = 2.7 × 10-7, *q* < 0.05) gene was significantly correlated with clinical improvements in treatment-resistant SCZ.
Nour El Huda et al., 2018	COMT CpG island of the 5′ upstream	138 SCZ 132 HC	Co-medications (NA)	Blood	Bisulfite conversion + PCR	• DNAm of COMT was negatively correlated with excitement (*p* < 0.001) and depressed (*p* = 0.001) scores
Miura et al., 2018	ANKK1 -162C to + 260C of the 5′ region	34 SCZ	Aripiprazole*(6 weeks)	Blood	Bisulfite conversion + PCR	• RES vs. NRES: no differences in DNAm levels at overall CpG sites; hypermethylation at CpG site 387 (*p* = 0.017) • DNAm at CpG sites 387 was negatively correlated with changes in total PANSS (*p* = 0.031), positive (*p* = 0.037) and negative scores (*p* = 0.039)
**Bipolar disorder**						
Burghardt et al., 2018	AKT1, AKT2, AKT3 Promoter region	30 BD	AAPs or MSs at least 3 months	FSM	PCR + MS-HRM	• AAPs patients: AKT2 DNAm and HOMA-IR were positively correlated (*p* = 0.3) • MSs patients: AKT2 DNAm and HOMA-IR was negatively correlated (*p* = 0.1)
**Major depressive disorder**						
Kang et al., 2013	5-HTT Promoter region	108 MDD	Ads (12 weeks)	Blood	Bisulfite conversion + PCR	• CpG site 2 and the average DNAm of 5-HTT were negatively correlated with changes of HAM-D scores (*p* < 0.05) • CpG sites 1 was negatively correlated with changes of HAMA scores (*p* < 0.05); • average DNAm was negatively correlated with changes of SOFAS scores (*p* < 0.05)
Powell et al., 2013	IL11 CpG island	113 MDD	Escitalopram or Nortriptyline (12 weeks)	Blood	Bisulfite conversion + PCR	• DNAm of CpG unit 5 was negatively correlated with changes of MADRS scores (*p* = 0.005, *q* = 0.055); • Escitalopram group: CpG unit 4 was positively correlated with changes of MADRS scores (*p* = 0.005, *q* = 0.055); • Nortriptyline group: CpG unit 4 was negatively correlated with changes of MADRS scores (*p* = 0.005, *q* = 0.055)
Tadić et al., 2014	BDNF Promoter region	39 MDD	Monoaminergic drugs (6 weeks)	Leus	NA	• RES vs. NRES: higher DNAm at CpG-87 of BDNF (*p* = 0.003, *q* = 0.03)
Okada et al., 2014	5-HTT CpG island	50 MDD 50 HC	ADs (6 weeks)	Blood	MCS	• DNAm of CpG 76 was positively correlated with total HAM-D scores (*p* = 0.03); • DNAm of CpG 3 was positively correlated with improvement ratio (HAM-D, *p* = 0.02)
Domschke et al., 2014	5-HTT Upstream of exon 1A	94 MDD	Escitalopram*(6 weeks)	Blood	Bisulfite sequencing	• CpG 1 (*p* = 0.048), CpG 2 (*p* = 0.002, *q* < = 0.05), CpG 4 (*p* = 0.029) and average DNAm (*p* = 0.005, *q* < = 0.05) of 5-HTT were positively correlated with changes of HAMD-21 scores
Domschke et al., 2015	MAO-A Promoter/exon1/intron1 regions	94 MDD	Escitalopram* (6 weeks)	Blood	Bisulfite sequencing	• Females: DNAm of CpG 1 (*p* = 0.04) and CpG 5 (*p* = 0.009) were positively correlated with changes of HAMD-21 scores; while average DNAm across all CpGs showed no association with treatment response • Males: no significant associations detected
Kahl et al., 2016	GLUT1/4 Promoter region	37 MDD	ADs (6 weeks)	Blood	Bisulfite sequencing	• REMs vs. NREMs: hypomethylation of the average DNAm of GLUT1 (*p* < 0.001); • No significant differences were observed for GLUT4 DNAm.
Iga et al., 2016	5-HTT Promoter region relate to TSS	28 MDD; 29 HC	ADs (8 weeks)	Leus	Bisulfite conversion + PCR	• DNAm of CpG 3 (*p* = 0.003) and CpG 5 (*p* = 0.004) were negatively correlated with baseline HAMD-17 scores • DNAm of CpG 2 (*p* = 0.04) was negatively correlated with clinical improvement as assessed with HAMD-17
Gassó et al., 2017	HTR1B Promoter region	57 MDD	Fluoxetine*(12 weeks)	Blood	Pyrosequencing	• A negative correlation was found between the average DNAm level and GAF/CGAS changes (*p* = 0.004)
Lieb et al., 2018	BDNF Exon IV or P11 promoter	561 MDD	Escitalopram*(8 weeks)	Blood	Bisulfite conversion + PCR	• DNAm status at CpG-87 of promoter exon IV (*p* = 0.029) was positively correlated with remission of the depressive symptomatology, especially in severe MDD patients (*n* = 199, *p* = 0.031)
Wang et al., 2018a	HTR1A/B promoter region	85 MDD	Escitalopram* (8 weeks)	Blood	Illumina Hiseq	REM VS. NREM: • Hypermethylation of CpG 668 (HTR1A, *p* = 0.025) and CpG 1401 (HTR1B, *p* = 0.033); • Higher DNAm changes after treatment in CpG 2793 (*p* = 0.015), CpG 2834 (*p* = 0.002), CpG 2927 (*p* = 0.023) and CpG 2937 (*p* = 0.003) of HTR1A and CpG-100 (*p* = 0.021) and CpG 1401 (*p* = 0.029) of HTR1B, compared with NREMs
Wang et al., 2018b	BDNF Promoter region	85 MDD	Escitalopram* (8 weeks)	Blood	Illumina Hiseq	• REM vs. NREM: hypermethylation of amplicon BDNF_1 (*p* = 0.006), BDNF_3 (*p* = 0.016), BDNF_4 (*p* = 0.029) and BDNF_5 (*p* = 0.036); • BDNF_1 (*p* = 0.004), BDNF_3 (*p* = 0.013), BDNF_4 (*p* = 0.034), BDNF_5 (*p* = 0.027) and average (*p* = 0.038) DNAm of BDNF was positively correlated with HAMD-17 changes
Wagner et al., 2019	BDNF Exon IV and P11 promoter	110 MDD	Escitalopram*(8 weeks)	Blood	Bisulfite conversion + PCR	• The BDNF exon IV promoter and P11 gene methylation did not predict a normalization of executive dysfunctions
Hsieh et al., 2019	BDNF Exon IX promoter	36 MDD	SSRIs (4 weeks)	Blood	Pyrosequencing	• RES vs. NRES: hypermethylation of CpG 24 (*p* = 0.029) and CpG 324 (*p* = 0.031) of BDNF promoter region
Draganov et al., 2019	IL6, IL6R, IL1-β CpG island relative to 5′ regulatory region	153 MDD	SSRIs (NA)	Blood	Bisulfite-pyrosequencing	• RES vs. NRES: hypermethylation of CpG IL6R_4 (*p* = 0.05)
Takeuchi et al., 2017	Whole genome	20 MDD	Paroxetine*(6 weeks)	Blood	HM450K	• BR vs. WR: 218 sites were nominally significant (*p* < 0.05), and 2 sites (PPFIA4 and HS3ST1) were significantly hypomethylated after FDR correction (*q* < 0.05).
Ju et al., 2019	Whole genome	177 MDD	Escitalopram (8 weeks)	Blood	EPIC	• RES vs. NRES: 303 sites were nominally significant (*p* < 0.05, Δβ ≥ ± 2%)
**Bipolar disorder & Major depressive disorder**						
Carlberg et al., 2014	BDNF Exon I promoter	207 MDD; 59 BD; 278 HC	ADs (NA)	Blood	Bisulfite conversion + PCR	• No significant correlations were found for DNAm levels and the total BDI sum score (available from *n* = 81 MDD)

Maudsley Staging Method, MSM; Responder, RES; Non-responder, NRES; Best-responder, BR; Worse-responder, WR; Methylation-sensitive High-resolution Melting, MS-HRM; Antipsychotics, APs; Atypical Antipsychotics, AAPs; Typical Antipsychotics, TAPs; Classic Antipsychotics, CAPs; Mood Stabilizers, MSs; Valproate, VPA; Lithium, Li; Fasting skeletal muscle, FSM. *Monotherapy was permitted for co-medications with other psycho-pharmacological agents or medications for insomnia.

Twenty-one candidate genes were evaluated and DNAm of 12 genes showed a significant correlation with clinical improvement (*p* < 0.05). Only BDNF (Carlberg et al., 2014; Tadić et al., 2014; Lieb et al., 2018; Wang et al., 2018b; Hsieh et al., 2019; Wagner et al., 2019), 5-HTT (Kang et al., 2013; Domschke et al., 2014; Okada et al., 2014; Iga et al., 2016), and HTR1B (Gassó et al., 2017; Wang et al., 2018a) were assessed in multiple MDD studies. No replication efforts were found in studies of other major psychiatric disorders. Baseline DNAm levels of BDNF in MDD patients in both blood and leukocytes was positively correlated with remission of depressive symptomatology after antidepressant treatment in 4 of 6 independent studies (Tadić et al., 2014; Lieb et al., 2018; Wang et al., 2018b; Hsieh et al., 2019).

Three studies used a genome-wide approach to evaluate the correlation between baseline DNAm and clinical improvement. Takeuchi et al. (2017) analyzed the changes of baseline DNAm between responders and non-responders of 20 paroxetine-treated MDD patients based on blood samples, using HM450K assays. Responders and non-responders had two CpG sites on genes PPFIA4 and HS3ST1 that were differently methylated (*q* < 0.05). Another study compared the DNAm changes in blood between responders and non-responders with 8 weeks of escitalopram treatment for 177 MDD patients, using the EPIC assays (Ju et al., 2019). They identified 303 (*p* < 0.05) sites with nominally significant differences between responders and non-responders, but none were significantly different after correction for multiple comparisons. Since these two drugs, paroxetine and escitalopram, are both SSRIs, we compared the reproducibility of the nominally significant results between these two studies. We found that none of the significant loci found to be associated with paroxetine could be replicated in the escitalopram treatment analysis. This failure at replication has to be considered in the context of differences in the platforms used, limitations in statistical power, and the 303 escitalopram-related sites detected by EPIC analysis were not included in HM450K assays. In evaluating whether results of candidate gene studies for MDD could be reproduced in genome-wide studies, we did not detect any overlap.

We did find one study involving schizophrenia, which evaluated the correlation between DNAm changes in blood after 1-year of clozapine treatment and changes of PANSS scores for 21 SCZ patients (Kinoshita et al., 2017). They found that DNAm in a site located at the CREBBP gene was negatively correlated with clinical improvement after multiple testing correction. However, this finding remains to be replicated. Further analysis showed that none of these significant candidate genes tested for antipsychotics (e.g., HTR1A, CYP3A4, COMT), especially clozapine, reached a nominal significance in genome-wide analysis.

### Comparison of DNAm Status Across Different Medications

Major medications for SCZ, BD, and MDD included antipsychotics, mood stabilizers and antidepressants. Antipsychotic medications are one of the primary treatments for the acute phases and the long-term prevention of recurrence for SCZ patients (Leucht et al., 2009; Hasan et al., 2017). Mood stabilizers, especially lithium, are a cornerstone in the long-term treatment of BD (Rybakowski, 2011; Geddes and Miklowitz, 2013). MDD patients are often treated with antidepressants (Drozda et al., 2014). However, the drug medications for each disorder are not mutually exclusive, as antipsychotic drugs may also be used in BD and MDD patients (Cruz et al., 2010; Patkar and Pae, 2013; Poo and Agius, 2014). The therapeutic efficacy of individual drugs is quite variable, and treatment trials to establish efficacy are laborious, so establishing biomarkers to improve guidance for treatment planning of individual patients is an important aim of psychiatric research. Predicting response to specific therapies could promote personalized medicine.

In this review, we evaluated 14 studies that compared DNAm after treatment with different medications, including 11 studies that used a candidate gene method and 3 that analyzed global methylation (Table 3). We found that antidepressant medication increased the DNAm status of promoter regions of the BDNF gene in both BD and MDD patients beyond the level seen in antidepressant-free patients. With advantages and disadvantages, it is worth noting that drug selection and treatment duration varied across studies.

**TABLE 3:** Summary of studies exploring comparison of DNAm status across different medications.

Studies	Genome Region analyzed	Sample size	treatment duration	Tissue	Platform	Main results
**Schizophrenia**						
Melas et al., 2012	Global methylation	129 SCZ; 171 HC	NA	Blood	LUMA	• Haloperidol vs. other APs: higher (control-like) global DNAm (*p* < 0.001)
Nour El Huda et al., 2018	COMT CpG islands	138 SCZ; 132 HC	NA	Blood	Bisulfite conversion + PCR	• AAP vs. TAP: lower DNAm status (*p* = 0.004); AAP vs. Sulpiride: lower DNAm status (*p* = 0.004) • Risperidone vs. TAP: lower DNAm status (*p* = 0.049)
**Bipolar disorder**						
D’Addario et al., 2012	BDNF Promoter region	49 BD I; 45 BD II; 52 HC	at least 1 months	PBMCs	Bisulfite conversion + RT-qPCR	• AD vs. AD-free: higher DNAm levels (*p* < 0.01, AD-free had a control-like DNAm levels); Li vs. other drugs: lower (control-like) DNAm levels (*p* < 0.05) • VPA vs. other drugs: lower (control-like) DNAm levels (*p* < 0.05)
Backlund et al., 2015	Global methylation	61 BD; 26 HC	at least 3 months	Blood	ELISA	• Li vs. HC: lower DNAm levels (*p* = 0.036) • Li + VPA vs. Li: higher DNAm levels (*p* = 0.011); • Li + AP vs. Li: higher DNAm levels (*p* = 0.071)
Burghardt et al., 2018	AKT1&AKT2 &AKT3 Promoter region	30 BD	at least 3 months	FSM	PCR + MS-HRM	• AAP vs. MSs: increase of AKT1 and AKT2 DNAm (*p* = 0.03; *p* = 0.02)
D’Addario et al., 2018	PDYN Promoter region	54 BD I; 45 BD II; 41 HC	at least 1 months	PBMCs	Bisulfite conversion + RT-qPCR	• Li or VPA vs. other drugs: lower (control-like) DNAm levels (*p* < 0.05)
Burghardt et al., 2019	Global methylation	28 BD; 13 HC	at least 3 months	FSM	LINE1	• AAP vs. HC: higher DNAm levels (*p* = 0.0004, *q* = 0.0048) • MSs vs. HC: higher DNAm levels (*p* = 0.12) • AAP vs. HC: higher DNAm levels (*p* = 0.0156, *q* = 0.037)
**Major depressive disorder**						
Ryan et al., 2017	IL6 Promoter region	92 MDD; 288 HC	4 waves of follow-up	Buccal swabs	SEQUENOM MassARRAY	• AD use was associated with a mean 4.6% increase in CpG2 DNAm of IL6 (*p* = 0.017)
**Schizophrenia & Bipolar disorder**						
Abdolmaleky et al., 2006	MB-COMT promoter region	40 SCZ; 35 BD; 40 HC	life times	Post-mortem brain	qMSP	• AP vs. drug-free: no differences • VPA did not specifically alter DNAm status
Abdolmaleky et al., 2011	HTR2A promoter region	35 SCZ; 35 BD; 35 HC	life times	Post-mortem brain	qMSP	• AP vs. drug-free: lower DNAm levels (*p* = 0.012) • drug-free vs. HC: higher DNAm levels in BD and SCZ and BD combined (*p* = 0.002 and *p* = 0.0027, respectively) • AAP vs. TAP: no differences
Abdolmaleky et al., 2014	5-HTT promoter region	35 SCZ; 35 BD; 35 HC	life times	Saliva; Post-mortem brain	qMSP	• AP vs. HC: no differences in both saliva and brain • drug-free vs. AP: higher DNAm levels in brain (*p* = 0.038) • drug-free vs. HC: higher DNAm levels in brain (*p* = 0.04)
Abdolmaleky et al., 2015	DTNBP1 Promoter flanking SP1 binding site	35 SCZ; 35 BD; 35 HC	life times	Saliva; Post-mortem brain	qMSP	• Drug-free vs. AP: higher DNAm status in BD (*p* = 0.045) • CAP vs. AAP + CAP: lower DNAm status in SCZ (*p* = 0.037) • CAP vs. AAP + CAP and AAP combined: decrease of DNAm status in SCZ (*p* = 0.017)
**Bipolar disorder & Major depressive disorder**						
Dell’Osso et al., 2014	BDNF Exon I promoter	43 MDD; 111 BD; 44 HC	NA	Blood	Bisulfite conversion + PCR	• Li or VPA vs. other drugs: tended to decrease (control-like) DNAm levels (no.sig)
Carlberg et al., 2014	BDNF Exon I promoter	207 MDD; 59 BD; 278 HC	NA	Blood	MS-qPCR	For 165 MDD: • AD vs. AD-free: higher DNAm levels (*p* = 0.0019) • AD vs. HC: higher DNAm levels (*p* < 0.0001) • AD-free vs. HC: no differences

*Luminometric Methylation Assay, LUMA; Methylation-sensitive High-resolution Melting, MS-HRM; Linear Interspersed Nuclear Element 1, LINE1; Enzyme-linked Immunosorbant Assay, ELISA; Antipsychotics, APs; Atypical Antipsychotics, AAPs; Typical Antipsychotics, TAPs; Classic Antipsychotics, CAPs; Mood Stabilizers, MSs; Valproate, VPA; Lithium, Li; Fasting skeletal muscle, FSM; Peripheral blood mononuclear cells, PBMCs; Quantitative methylation specific PCR, qMSP.*

## Challenges and Future Directions

### Underpowered Studies — Large Cohort Collaborations Are Needed

Insufficient statistical power is a major challenge for pharmacoepigenetic studies in psychiatric disorders. The cost of using methylation panels, the high cost of clinical trials where drug therapy is controlled along with diverse drugs used across studies, and the large number of sites for methylation investigation, together conspire to limit sample sizes and statistical power to detect effects of interest. These factors limit our ability to evaluate reproducibility of current pharmacoepigenetic findings. Although several interesting associations between DNAm and drug treatment outcomes occurred in relatively small sample sizes, findings from these studies should be considered preliminary until replication in a larger sample size is pursued. So, given that collecting clinical data with a large-sample is challenging and costly, small-sample studies remain the focus of most investigators in this area via hypothesis-generating studies.

To fill this gap, multi-site collaborations with ethnically diverse groups are needed in studies of the pharmacoepigenetics of psychiatric disorders. Prominent consortia have been established which looked at pharmacogenomics in psychiatric disorders, like the STAR^∗^D project (Rush et al., 2004), the Genome-based Therapeutic Drugs for Depression (GENDEP) project (Uher et al., 2010), the Chinese Antipsychotics Pharmacogenomics Consortium (CAPC) (Yu et al., 2018), and others. These consortia benefit ongoing and future pharmacoepigenetic studies by providing preliminary findings that can be examined in new samples. For example, Powell et al. (2013) found that DNAm in IL11 could predict the clinical response to antidepressants using samples from the GENDEP project. Large international consortia, as have been developed for genetic association studies, are needed to study adequate numbers of patients with samples collected using a uniform procedure and with standardized drug treatment.

### Recommendation for Clinical Design: Drug Selection, Treatment Duration, Evaluation, and Tissue Selection for Pharmacoepigenetic Studies

Neuropsychiatric drugs are grouped into various classes with slightly different mechanisms of action. It is clear that from an efficacy perspective, first line treatments for all these disorders leaves considerable room for improvement, potentially in new drug development and in strategies for personalized medicine (Leucht et al., 2012; Cipriani et al., 2018; Huhn et al., 2019; Pillinger et al., 2020). Identification of new biomarkers for these disorders is difficult, primarily because of the lack of knowledge about disease pathophysiology and mechanisms of drug action.

A major aim of psychopharmacology research is to develop valid strategies for selecting the right drug with the right dose for the “right” patients to optimize outcomes with low risks of side effects and relapse. Prior work is limited in supporting progress in this area. Across the published studies in this area, the types of medications were not uniform for each disorder, making comparison of studies difficult. Only 8% of studies used monotherapy for patients. While 27% of studies claimed to use monotherapy for patients, but we noticed that besides the same primary medication, they temporarily permitted secondary medications (e.g., benzodiazepines, flunitrazepam) for insomnia or some side effects, which themselves can also exert epigenetic effects.

Choice of duration for drug treatment trials is also important, yet variable across prior work. Treatment-resistance, non-adherence, late-emerging side effects and relapse are frequent events in clinical practice, some of which are seen only in longer term protocol participation. Aside from these factors, DNAm status is dynamic and can be affected by various environmental events during treatment that are unrelated to drug effects (Bjornsson et al., 2008).

Definition of treatment outcomes for psychiatric disorders is also a challenge, as different approaches may be used across studies. A recent study suggested that integrating genomics and phenotypic measures data could increase the accuracy of prediction of drug response (Kauppi et al., 2018). Thus, future research should consider an integrated examination of genetic, DNAm and dense phenotype assessment of drug effects not only with behavioral ratings but with direct *in vivo* assessment of brain physiology and treatment (Huang et al., 2019). Notably, all of these prediction models need to be covered across the relevant age span, ethnic groups, and sexes.

Predictors of drug response for psychiatric disorders ideally need to have biological relevance based on a solid mechanistic understanding of the pathophysiology of brain dysfunction. Most of the pharmacoepigenetic studies utilized clinically accessible tissues such as blood, saliva, and others. However, whether biomarkers identified in these tissues could reflect similar DNAm changes in the brain needs further study. Several brain banks with post-mortem tissue from patients that had neuropsychiatric disorders have been established, which generated big data sets at multiple regulatory levels (e.g., DNAm, gene expression, and other omics) (Wang et al., 2019). These multidimensional data sets could facilitate the exploration of the peripheral-brain relationship. Since epigenetic modifications are known to fluctuate over the lifespan, resolving the causal relationships in the epigenetic study of drug response using postmortem brains will be challenging. Cellular models will likely fill the gaps where both peripheral tissue and postmortem brain will not be able to deliver the answers.

### System-Wide Analysis Could Benefit Pharmacoepigenetic Studies

Candidate gene studies contributed to answering specific questions in biology, but they do not provide system-wide information. In this review, we noticed that the assessed candidate genes were generally not the top signals in genome-wide studies, and could not even reach a genome-wide significance. The genes that change the most between pre- and post-treatment, or are most strongly associated with clinical improvement, remain to be discovered. Also, pathway-based research rather than gene-based approaches may be more efficient. Different genes that converge on the same pathway may each contribute modestly, but together may robustly co-influence the functional abnormality. Therefore, system-wide research could provide better biomarkers than individual candidate genes.

Regulation of drug responses occurs at various levels including genetics, epigenetics, transcriptional, and protein modification, and also involves many functional pathways (Amare et al., 2017). To date, the underlying system of drug response-related DNAm remains poorly understood. Some have proposed that one or more components involved in a pathway, even if not risk genes, may be a better drug target than risk genes themselves (Wang et al., 2010; Harrison, 2015). One study assessed the interaction between genetic variates with DNAm for the IL-11 gene, which identified a possible regulatory relationship that could be used to predict antidepressant response for MDD patients (Powell et al., 2013). However, studies to date only scratched the surface of the complex regulatory system of drug response. Systematic integration of genomic data and genome-wide DNAm data will be more effective and unbiased for learning about these regulatory processes, hopefully leading to the discovery of underlying drug response networks.

### Data Analysis for Pharmacoepigenetic Studies

Strict statistical criteria and sufficient attention to covariances are critical for reducing false-positive rates. DNAm status is not a constant like DNA sequence are therefore is subject to influences from more confounders. Further, approaches for DNAm assessment can be easily confounded by technical artifacts. DNAm levels are also affected by factors including age (Horvath, 2013; Jaffe et al., 2016; Merid et al., 2020), sex (Maschietto et al., 2017; Xia et al., 2019), circadian rhythms (Lim et al., 2014), smoking (Shenker et al., 2013), drinking (Philibert et al., 2012), and others. All of these considerations for study design and data analysis need to meet standard requirements for drug response-related DNAm studies as in most genomic studies.

Methylation profiling could be easily confounded by batch effects. Systematic error can be introduced when samples are processed in multiple batches (e.g., the same sample measured at different times), which cannot be eliminated unless all samples are run in a single batch (Chen et al., 2011). It is especially important that cases and controls not be put in separate batches. Except for batch effects, positional effects also exist in the microarray and bias analysis (Jiao et al., 2018). Positional effects are emerging when the same sample in different physical positions on the array and could bias methylation levels and lead to false findings. From our review, we observed that only one study did the batch effects correction (Ju et al., 2019), and none of these studies corrected for positional effects according to the method description in those papers. We cannot rule out the possibility that the data were properly processed but failed to be reported in the papers, but not reporting such details at least indicated the lack of attention to the serious issues.

Considering the influence of demographic information (e.g., age, sex, BMI) is also important. In this review, nearly 49% of studies did not control for covariates in their analyses. When there was a correction for covariates, typically only sex and age was controlled. Since DNAm is highly cell-type-specific, cellular heterogeneity of blood may skew DNAm patterns, influencing findings in drug response for psychiatric disorders (You et al., 2020). All studies did not control cell-type compositions. Collecting data about potentially useful covariates, and controlling for them in analyses, should be standard practice in future research for drug response studies. However, we note that large samples will be needed to develop appropriate modeling for covariate effects, as a few outliers or complex covariate interactions can exert effects that are challenging to deal with in smaller sample studies.

Additionally, strict and consistent statistical criteria for defining significance need to be applied that correct for multiple comparisons. Most published studies considered a nominal *p* < 0.05 to be the threshold for statistical significance, even when multiple genes or CpG sites were tested. We noticed that only 59% of the previous studies did the correction for multiple testing. False-positive results likely occurred in published work (Joober et al., 2012).

### Summary

In this review, we found that only DNAm of BDNF was consistently related to clinical outcomes, an effect observed in four independent studies of MDD patients. Although pharmacoepigenetic studies to date are still limited in number and sample size, preliminary studies to date that evaluated changes in drug response-related DNAm across major psychiatric disorders provide evidence for an intriguing possibility of predictive DNAm biomarkers for drug treatment. A system-wide study using strict analytical and statistical procedures in large multisite studies may best advance identification of useful DNAm predictors for drug response in psychiatric disorders.

## Author Contributions

JZ, ML, XW, and YH did the manuscript collection. JZ did the manuscript writing. JZ and ML designed and generated the manuscript figures and tables. YX, JS, and RK helped with manuscript writing. CC and CL helped with study design and manuscript writing. All authors contributed to the article and approved the submitted version.

## Conflict of Interest

The authors declare that the research was conducted in the absence of any commercial or financial relationships that could be construed as a potential conflict of interest.

## References

[B1] Aaronson S. T. Sears P. Ruvuna F. Bunker M. Conway C. R. Dougherty D. D. (2017). A 5-year observational study of patients with treatment-resistant depression treated with vagus nerve stimulation or treatment as usual: comparison of response, remission, and suicidality. *Am. J. Psychiatry* 174 640–648. 10.1176/appi.ajp.2017.16010034 28359201

[B2] Abdolmaleky H. M. Cheng K. H. Faraone S. V. Wilcox M. Glatt S. J. Gao F. (2006). Hypomethylation of MB-COMT promoter is a major risk factor for schizophrenia and bipolar disorder. *Hum. Mol. Genet.* 15 3132–3145. 10.1093/hmg/ddl253 16984965 PMC2799943

[B3] Abdolmaleky H. M. Nohesara S. Ghadirivasfi M. Lambert A. W. Ahmadkhaniha H. Ozturk S. (2014). DNA hypermethylation of serotonin transporter gene promoter in drug naïve patients with schizophrenia. *Schizophr. Res.* 152 373–380. 10.1016/j.schres.2013.12.007 24411530 PMC7863587

[B4] Abdolmaleky H. M. Pajouhanfar S. Faghankhani M. Joghataei M. T. Mostafavi A. Thiagalingam S. (2015). Antipsychotic drugs attenuate aberrant DNA methylation of DTNBP1 (dysbindin) promoter in saliva and post-mortem brain of patients with schizophrenia and Psychotic bipolar disorder. *Am. J. Med. Genet. B Neuropsychiatr. Genet.* 168 687–696. 10.1002/ajmg.b.32361 26285059

[B5] Abdolmaleky H. M. Yaqubi S. Papageorgis P. Lambert A. W. Ozturk S. Sivaraman V. (2011). Epigenetic dysregulation of HTR2A in the brain of patients with schizophrenia and bipolar disorder. *Schizophr. Res.* 129 183–190. 10.1016/j.schres.2011.04.007 21550210

[B6] Amare A. T. Schubert K. O. Baune B. T. (2017). Pharmacogenomics in the treatment of mood disorders: strategies and opportunities for personalized psychiatry. *EPMA J.* 8 211–227. 10.1007/s13167-017-0112-8 29021832 PMC5607053

[B7] Backlund L. Wei Y. B. Martinsson L. Melas P. A. Liu J. J. Mu N. (2015). Mood stabilizers and the influence on global leukocyte DNA methylation in bipolar disorder. *Mol. Neuropsychiatry* 1 76–81. 10.1159/000430867 27602359 PMC4996001

[B8] Barros-Silva D. Marques C. J. Henrique R. Jerónimo C. (2018). Profiling DNA methylation based on next-generation sequencing approaches: new insights and clinical applications. *Genes (Basel)* 9:429. 10.3390/genes9090429 30142958 PMC6162482

[B9] Baum A. E. Akula N. Cabanero M. Cardona I. Corona W. Klemens B. (2008). A genome-wide association study implicates diacylglycerol kinase eta (DGKH) and several other genes in the etiology of bipolar disorder. *Mol. Psychiatry* 13 197–207. 10.1038/sj.mp.4002012 17486107 PMC2527618

[B10] Bibikova M. Le J. Barnes B. Saedinia-Melnyk S. Zhou L. Shen R. (2009). Genome-wide DNA methylation profiling using Infinium^®^ assay. *Epigenomics* 1 177–200. 10.2217/epi.09.14 22122642

[B11] Bjornsson H. T. Sigurdsson M. I. Fallin M. D. Irizarry R. A. Aspelund T. Cui H. (2008). Intra-individual change over time in DNA methylation with familial clustering. *JAMA* 299 2877–2883. 10.1001/jama.299.24.2877 18577732 PMC2581898

[B12] Brandl E. J. Tiwari A. K. Zai C. C. Nurmi E. L. Chowdhury N. I. Arenovich T. (2016). Genome-wide association study on antipsychotic-induced weight gain in the CATIE sample. *Pharmacogenomics J.* 16 352–356. 10.1038/tpj.2015.59 26323598

[B13] Braun P. R. Han S. Hing B. Nagahama Y. Gaul L. N. Heinzman J. T. (2019). Genome-wide DNA methylation comparison between live human brain and peripheral tissues within individuals. *Transl. Psychiatry* 9:47. 10.1038/s41398-019-0376-y 30705257 PMC6355837

[B14] Burghardt K. J. Howlett B. H. Sanders E. Dass S. E. Msallaty Z. Mallisho A. (2019). Skeletal muscle DNA methylation modifications and psychopharmacologic treatment in bipolar disorder. *Eur. Neuropsychopharmacol.* 29 1365–1373. 10.1016/j.euroneuro.2019.10.001 31635791 PMC6924624

[B15] Burghardt K. J. Khoury A. S. Msallaty Z. Yi Z. Seyoum B. (2020). Antipsychotic medications and DNA Methylation in schizophrenia and bipolar disorder: a systematic review. *Pharmacotherapy* 40 331–342. 10.1002/phar.2375 32058614 PMC7152563

[B16] Burghardt K. J. Seyoum B. Dass S. E. Sanders E. Mallisho A. Yi Z. (2018). Association of protein kinase B (AKT) DNA hypermethylation with maintenance atypical antipsychotic treatment in patients with bipolar disorder. *Pharmacotherapy* 38 428–435. 10.1002/phar.2097 29484683 PMC5999031

[B17] Carlberg L. Scheibelreiter J. Hassler M. R. Schloegelhofer M. Schmoeger M. Ludwig B. (2014). Brain-derived neurotrophic factor (BDNF)-epigenetic regulation in unipolar and bipolar affective disorder. *J. Affect. Disord.* 168 399–406. 10.1016/j.jad.2014.07.022 25106037

[B18] Chan T. A. Baylin S. B. (2012). Epigenetic biomarkers. *Curr. Top. Microbiol. Immunol.* 355 189–216. 10.1007/82_2011_165 21818705

[B19] Chen C. Grennan K. Badner J. Zhang D. Gershon E. Jin L. (2011). Removing batch effects in analysis of expression microarray data: an evaluation of six batch adjustment methods. *PLoS One* 6:e17238. 10.1371/journal.pone.0017238 21386892 PMC3046121

[B20] Chen C. Zhang C. Cheng L. Reilly J. L. Bishop J. R. Sweeney J. A. (2014). Correlation between DNA methylation and gene expression in the brains of patients with bipolar disorder and schizophrenia. *Bipolar Disord.* 16 790–799. 10.1111/bdi.12255 25243493 PMC4302408

[B21] Cipriani A. Furukawa T. A. Salanti G. Chaimani A. Atkinson L. Z. Ogawa Y. (2018). Comparative efficacy and acceptability of 21 antidepressant drugs for the acute treatment of adults with major depressive disorder: a systematic review and network meta-analysis. *Lancet* 391 1357–1366. 10.1016/s0140-6736(17)32802-7 29477251 PMC5889788

[B22] Couldrey C. Cave V. (2014). Assessing DNA methylation levels in animals: choosing the right tool for the job. *Anim. Genet.* 45(Suppl. 1) 15–24. 10.1111/age.12186 24990588

[B23] Cruz N. Sanchez-Moreno J. Torres F. Goikolea J. M. Valentí M. Vieta E. (2010). Efficacy of modern antipsychotics in placebo-controlled trials in bipolar depression: a meta-analysis. *Int. J. Neuropsychopharmacol.* 13 5–14. 10.1017/s1461145709990344 19638254

[B24] D’Addario C. Dell’Osso B. Palazzo M. C. Benatti B. Lietti L. Cattaneo E. (2012). Selective DNA methylation of BDNF promoter in bipolar disorder: differences among patients with BDI and BDII. *Neuropsychopharmacology* 37 1647–1655. 10.1038/npp.2012.10 22353757 PMC3358733

[B25] D’Addario C. Palazzo M. C. Benatti B. Grancini B. Pucci M. Di Francesco A. (2018). Regulation of gene transcription in bipolar disorders: role of DNA methylation in the relationship between prodynorphin and brain derived neurotrophic factor. *Prog. Neuropsychopharmacol. Biol. Psychiatry* 82 314–321. 10.1016/j.pnpbp.2017.08.011 28830794 PMC5859566

[B26] Davies M. N. Volta M. Pidsley R. Lunnon K. Dixit A. Lovestone S. (2012). Functional annotation of the human brain methylome identifies tissue-specific epigenetic variation across brain and blood. *Genome Biol.* 13:R43. 10.1186/gb-2012-13-6-r43 22703893 PMC3446315

[B27] Dedeurwaerder S. Defrance M. Bizet M. Calonne E. Bontempi G. Fuks F. (2014). A comprehensive overview of infinium humanmethylation450 data processing. *Brief Bioinform.* 15 929–941. 10.1093/bib/bbt054 23990268 PMC4239800

[B28] Dedeurwaerder S. Defrance M. Calonne E. Denis H. Sotiriou C. Fuks F. (2011). Evaluation of the infinium methylation 450K technology. *Epigenomics* 3 771–784. 10.2217/epi.11.105 22126295

[B29] Dell’Osso B. D’Addario C. Carlotta Palazzo M. Benatti B. Camuri G. Galimberti D. (2014). Epigenetic modulation of BDNF gene: differences in DNA methylation between unipolar and bipolar patients. *J. Affect. Disord.* 166 330–333. 10.1016/j.jad.2014.05.020 25012449

[B30] Domschke K. Tidow N. Schwarte K. Deckert J. Lesch K. P. Arolt V. (2014). Serotonin transporter gene hypomethylation predicts impaired antidepressant treatment response. *Int. J. Neuropsychopharmacol.* 17 1167–1176. 10.1017/s146114571400039x 24679990

[B31] Domschke K. Tidow N. Schwarte K. Ziegler C. Lesch K. P. Deckert J. (2015). Pharmacoepigenetics of depression: no major influence of MAO-A DNA methylation on treatment response. *J. Neural Transm.* 122 99–108. 10.1007/s00702-014-1227-x 24809685

[B32] Dong E. Nelson M. Grayson D. R. Costa E. Guidotti A. (2008). Clozapine and sulpiride but not haloperidol or olanzapine activate brain DNA demethylation. *Proc. Natl. Acad. Sci. U.S.A.* 105 13614–13619. 10.1073/pnas.0805493105 18757738 PMC2533238

[B33] Draganov M. Arranz M. J. Salazar J. de Diego-Adeliño J. Gallego-Fabrega C. Jubero M. (2019). Association study of polymorphisms within inflammatory genes and methylation status in treatment response in major depression. *Eur. Psychiatry* 60 7–13. 10.1016/j.eurpsy.2019.05.003 31100612

[B34] Drozda K. Müller D. J. Bishop J. R. (2014). Pharmacogenomic testing for neuropsychiatric drugs: current status of drug labeling, guidelines for using genetic information, and test options. *Pharmacotherapy* 34 166–184. 10.1002/phar.1398 24523097 PMC3939793

[B35] Gassó P. Rodríguez N. Blázquez A. Monteagudo A. Boloc D. Plana M. T. (2017). Epigenetic and genetic variants in the HTR1B gene and clinical improvement in children and adolescents treated with fluoxetine. *Prog. Neuropsychopharmacol. Biol. Psychiatry* 75 28–34. 10.1016/j.pnpbp.2016.12.003 28025020

[B36] Gaudet F. Rideout W. M. III Meissner A. Dausman J. Leonhardt H. Jaenisch R. (2004). Dnmt1 expression in pre- and postimplantation embryogenesis and the maintenance of IAP silencing. *Mol. Cell. Biol.* 24 1640–1648. 10.1128/mcb.24.4.1640-1648.2004 14749379 PMC344181

[B37] GBD 2015 Disease and Injury Incidence and Prevalence Collaborators (2016). Global, regional, and national incidence, prevalence, and years lived with disability for 310 diseases and injuries, 1990-2015: a systematic analysis for the global burden of disease study 2015. *Lancet* 388 1545–1602. 10.1016/s0140-6736(16)31678-6 27733282 PMC5055577

[B38] Geddes J. R. Miklowitz D. J. (2013). Treatment of bipolar disorder. *Lancet* 381 1672–1682. 10.1016/s0140-6736(13)60857-0 23663953 PMC3876031

[B39] Goud Alladi C. Etain B. Bellivier F. Marie-Claire C. (2018). DNA methylation as a biomarker of treatment response variability in serious mental illnesses: a systematic review focused on bipolar disorder, schizophrenia, and major depressive disorder. *Int. J. Mol. Sci.* 19:3026. 10.3390/ijms19103026 30287754 PMC6213157

[B40] Guintivano J. Aryee M. J. Kaminsky Z. A. (2013). A cell epigenotype specific model for the correction of brain cellular heterogeneity bias and its application to age, brain region and major depression. *Epigenetics* 8 290–302. 10.4161/epi.23924 23426267 PMC3669121

[B41] Guo J. U. Su Y. Shin J. H. Shin J. Li H. Xie B. (2014). Distribution, recognition and regulation of non-CpG methylation in the adult mammalian brain. *Nat. Neurosci.* 17 215–222. 10.1038/nn.3607 24362762 PMC3970219

[B42] Harrison P. J. (2015). The current and potential impact of genetics and genomics on neuropsychopharmacology. *Eur. Neuropsychopharmacol.* 25 671–681. 10.1016/j.euroneuro.2013.02.005 23528807

[B43] Hasan A. Falkai P. Wobrock T. Lieberman J. Glenthøj B. Gattaz W. F. (2017). World federation of societies of biological psychiatry (WFSBP) guidelines for biological treatment of schizophrenia – a short version for primary care. *Int. J. Psychiatry Clin. Pract.* 21 82–90. 10.1080/13651501.2017.1291839 28498090

[B44] Heerboth S. Lapinska K. Snyder N. Leary M. Rollinson S. Sarkar S. (2014). Use of epigenetic drugs in disease: an overview. *Genet. Epigenet.* 6 9–19. 10.4137/geg.S12270 25512710 PMC4251063

[B45] Hellman A. Chess A. (2007). Gene body-specific methylation on the active X chromosome. *Science* 315 1141–1143. 10.1126/science.1136352 17322062

[B46] Holliday R. Pugh J. E. (1975). DNA modification mechanisms and gene activity during development. *Science* 187 226–232. 1111098

[B47] Horvath S. (2013). DNA methylation age of human tissues and cell types. *Genome Biol.* 14:R115. 10.1186/gb-2013-14-10-r115 24138928 PMC4015143

[B48] Horvath S. Zhang Y. Langfelder P. Kahn R. S. Boks M. P. van Eijk K. (2012). Aging effects on DNA methylation modules in human brain and blood tissue. *Genome Biol.* 13:R97. 10.1186/gb-2012-13-10-r97 23034122 PMC4053733

[B49] Hsieh M. T. Lin C. C. Lee C. T. Huang T. L. (2019). Abnormal brain-derived neurotrophic factor exon IX promoter methylation, protein, and mRNA levels in patients with major depressive disorder. *J. Clin. Med.* 8:568. 10.3390/jcm8050568 31027379 PMC6571872

[B50] Huang X. Gong Q. Sweeney J. A. Biswal B. B. (2019). Progress in psychoradiology, the clinical application of psychiatric neuroimaging. *Br. J. Radiol.* 92:20181000. 10.1259/bjr.20181000 31170803 PMC6732936

[B51] Huhn M. Nikolakopoulou A. Schneider-Thoma J. Krause M. Samara M. Peter N. (2019). Comparative efficacy and tolerability of 32 oral antipsychotics for the acute treatment of adults with multi-episode schizophrenia: a systematic review and network meta-analysis. *Lancet* 394 939–951. 10.1016/s0140-6736(19)31135-3 31303314 PMC6891890

[B52] Hutnick L. K. Huang X. Loo T. C. Ma Z. Fan G. (2010). Repression of retrotransposal elements in mouse embryonic stem cells is primarily mediated by a DNA methylation-independent mechanism. *J. Biol. Chem.* 285 21082–21091. 10.1074/jbc.M110.125674 20404320 PMC2898347

[B53] Hyman S. E. (2012). Revolution stalled. *Sci. Transl. Med.* 4:155cm111. 10.1126/scitranslmed.3003142 23052291

[B54] Iga J. Watanabe S. Y. Numata S. Umehara H. Nishi A. Kinoshita M. (2016). Association study of polymorphism in the serotonin transporter gene promoter, methylation profiles, and expression in patients with major depressive disorder. *Hum. Psychopharmacol.* 31 193–199. 10.1002/hup.2527 27005686

[B55] Jaffe A. E. Gao Y. Deep-Soboslay A. Tao R. Hyde T. M. Weinberger D. R. (2016). Mapping DNA methylation across development, genotype and schizophrenia in the human frontal cortex. *Nat. Neurosci.* 19 40–47. 10.1038/nn.4181 26619358 PMC4783176

[B56] Jiao C. Zhang C. Dai R. Xia Y. Wang K. Giase G. (2018). Positional effects revealed in Illumina methylation array and the impact on analysis. *Epigenomics* 10 643–659. 10.2217/epi-2017-0105 29469594 PMC6021926

[B57] Jones P. A. (2012). Functions of DNA methylation: islands, start sites, gene bodies and beyond. *Nat. Rev. Genet.* 13 484–492. 10.1038/nrg3230 22641018

[B58] Joober R. Schmitz N. Annable L. Boksa P. (2012). Publication bias: what are the challenges and can they be overcome? *J. Psychiatry Neurosci.* 37 149–152. 10.1503/jpn.120065 22515987 PMC3341407

[B59] Ju C. Fiori L. M. Belzeaux R. Theroux J. F. Chen G. G. Aouabed Z. (2019). Integrated genome-wide methylation and expression analyses reveal functional predictors of response to antidepressants. *Transl. Psychiatry* 9:254. 10.1038/s41398-019-0589-0 31594917 PMC6783543

[B60] Kahl K. G. Georgi K. Bleich S. Muschler M. Hillemacher T. Hilfiker-Kleinert D. (2016). Altered DNA methylation of glucose transporter 1 and glucose transporter 4 in patients with major depressive disorder. *J. Psychiatr. Res.* 76 66–73. 10.1016/j.jpsychires.2016.02.002 26919485

[B61] Kaminsky Z. Tochigi M. Jia P. Pal M. Mill J. Kwan A. (2012). A multi-tissue analysis identifies HLA complex group 9 gene methylation differences in bipolar disorder. *Mol. Psychiatry* 17 728–740. 10.1038/mp.2011.64 21647149

[B62] Kang H. J. Kim J. M. Stewart R. Kim S. Y. Bae K. Y. Kim S. W. (2013). Association of SLC6A4 methylation with early adversity, characteristics and outcomes in depression. *Prog. Neuropsychopharmacol. Biol. Psychiatry* 44 23–28. 10.1016/j.pnpbp.2013.01.006 23333376

[B63] Katz A. J. Dusetzina S. B. Farley J. F. Ellis A. R. Gaynes B. N. Castillo W. C. (2012). Distressing adverse events after antidepressant switch in the Sequenced Treatment Alternatives to Relieve Depression (STAR^∗^D) trial: influence of adverse events during initial treatment with citalopram on development of subsequent adverse events with an alternative antidepressant. *Pharmacotherapy* 32 234–243. 10.1002/j.1875-9114.2011.01020.x 22392456

[B64] Kauppi K. Fan C. C. McEvoy L. K. Holland D. Tan C. H. Chen C. H. (2018). Combining polygenic hazard score with volumetric MRI and cognitive measures improves prediction of progression from mild cognitive impairment to Alzheimer’s disease. *Front. Neurosci.* 12:260. 10.3389/fnins.2018.00260 29760643 PMC5937163

[B65] Kinoshita M. Numata S. Tajima A. Yamamori H. Yasuda Y. Fujimoto M. (2017). Effect of clozapine on DNA methylation in peripheral leukocytes from patients with treatment-resistant schizophrenia. *Int. J. Mol. Sci.* 18:632. 10.3390/ijms18030632 28335437 PMC5372645

[B66] Kubota T. Miyake K. Hirasawa T. (2012). Epigenetic understanding of gene-environment interactions in psychiatric disorders: a new concept of clinical genetics. *Clin. Epigenetics* 4:1. 10.1186/1868-7083-4-1 22414323 PMC3305338

[B67] Kurdyukov S. Bullock M. (2016). DNA methylation analysis: choosing the right method. *Biology (Basel)* 5;3. 10.3390/biology5010003 26751487 PMC4810160

[B68] Leucht S. Corves C. Arbter D. Engel R. R. Li C. Davis J. M. (2009). Second-generation versus first-generation antipsychotic drugs for schizophrenia: a meta-analysis. *Lancet* 373 31–41. 10.1016/s0140-6736(08)61764-x 19058842

[B69] Leucht S. Tardy M. Komossa K. Heres S. Kissling W. Salanti G. (2012). Antipsychotic drugs versus placebo for relapse prevention in schizophrenia: a systematic review and meta-analysis. *Lancet* 379 2063–2071. 10.1016/s0140-6736(12)60239-6 22560607

[B70] Liberati A. Altman D. G. Tetzlaff J. Mulrow C. Gøtzsche P. C. Ioannidis J. P. (2009). The PRISMA statement for reporting systematic reviews and meta-analyses of studies that evaluate healthcare interventions: explanation and elaboration. *BMJ* 339:b2700. 10.1136/bmj.b2700 19622552 PMC2714672

[B71] Lieb K. Dreimüller N. Wagner S. Schlicht K. Falter T. Neyazi A. (2018). BDNF plasma levels and BDNF exon IV promoter methylation as predictors for antidepressant treatment response. *Front. Psychiatry* 9:511. 10.3389/fpsyt.2018.00511 30459647 PMC6232909

[B72] Lim A. S. Srivastava G. P. Yu L. Chibnik L. B. Xu J. Buchman A. S. (2014). 24-hour rhythms of DNA methylation and their relation with rhythms of RNA expression in the human dorsolateral prefrontal cortex. *PLoS Genet.* 10:e1004792. 10.1371/journal.pgen.1004792 25375876 PMC4222754

[B73] Lisoway A. J. Zai C. C. Tiwari A. K. Kennedy J. L. (2018). DNA methylation and clinical response to antidepressant medication in major depressive disorder: a review and recommendations. *Neurosci. Lett.* 669 14–23. 10.1016/j.neulet.2016.12.071 28063933

[B74] Lister R. Mukamel E. A. Nery J. R. Urich M. Puddifoot C. A. Johnson N. D. (2013). Global epigenomic reconfiguration during mammalian brain development. *Science* 341:1237905. 10.1126/science.1237905 23828890 PMC3785061

[B75] Liu C. Jiao C. Wang K. Yuan N. (2018). DNA methylation and psychiatric disorders. *Prog. Mol. Biol. Transl. Sci.* 157 175–232. 10.1016/bs.pmbts.2018.01.006 29933950

[B76] Lowe R. Gemma C. Beyan H. Hawa M. I. Bazeos A. Leslie R. D. (2013). Buccals are likely to be a more informative surrogate tissue than blood for epigenome-wide association studies. *Epigenetics* 8 445–454. 10.4161/epi.24362 23538714 PMC3674053

[B77] Maschietto M. Bastos L. C. Tahira A. C. Bastos E. P. Euclydes V. L. Brentani A. (2017). Sex differences in DNA methylation of the cord blood are related to sex-bias psychiatric diseases. *Sci. Rep.* 7:44547. 10.1038/srep44547 28303968 PMC5355991

[B78] Masliah E. Dumaop W. Galasko D. Desplats P. (2013). Distinctive patterns of DNA methylation associated with Parkinson disease: identification of concordant epigenetic changes in brain and peripheral blood leukocytes. *Epigenetics* 8 1030–1038. 10.4161/epi.25865 23907097 PMC3891683

[B79] Melas P. A. Rogdaki M. Ösby U. Schalling M. Lavebratt C. Ekström T. J. (2012). Epigenetic aberrations in leukocytes of patients with schizophrenia: association of global DNA methylation with antipsychotic drug treatment and disease onset. *Faseb J.* 26 2712–2718. 10.1096/fj.11-202069 22426120

[B80] Merid S. K. Novoloaca A. Sharp G. C. Küpers L. K. Kho A. T. Roy R. (2020). Epigenome-wide meta-analysis of blood DNA methylation in newborns and children identifies numerous loci related to gestational age. *Genome Med.* 12:25. 10.1186/s13073-020-0716-9 32114984 PMC7050134

[B81] Mill J. Petronis A. (2007). Molecular studies of major depressive disorder: the epigenetic perspective. *Mol. Psychiatry* 12 799–814. 10.1038/sj.mp.4001992 17420765

[B82] Mill J. Tang T. Kaminsky Z. Khare T. Yazdanpanah S. Bouchard L. (2008). Epigenomic profiling reveals DNA-methylation changes associated with major psychosis. *Am. J. Hum. Genet.* 82 696–711. 10.1016/j.ajhg.2008.01.008 18319075 PMC2427301

[B83] Miura I. Kunii Y. Hino M. Hoshino H. Matsumoto J. Kanno-Nozaki K. (2018). DNA methylation of ANKK1 and response to aripiprazole in patients with acute schizophrenia: a preliminary study. *J. Psychiatr. Res.* 100 84–87. 10.1016/j.jpsychires.2018.02.018 29499474

[B84] Moore L. D. Le T. Fan G. (2013). DNA methylation and its basic function. *Neuropsychopharmacology* 38 23–38. 10.1038/npp.2012.112 22781841 PMC3521964

[B85] Moore M. Yuen H. M. Dunn N. Mullee M. A. Maskell J. Kendrick T. (2009). Explaining the rise in antidepressant prescribing: a descriptive study using the general practice research database. *BMJ* 339:b3999. 10.1136/bmj.b3999 19833707 PMC2762496

[B86] Moran S. Arribas C. Esteller M. (2016). Validation of a DNA methylation microarray for 850,000 CpG sites of the human genome enriched in enhancer sequences. *Epigenomics* 8 389–399. 10.2217/epi.15.114 26673039 PMC4864062

[B87] Nelson M. R. Johnson T. Warren L. Hughes A. R. Chissoe S. L. Xu C. F. (2016). The genetics of drug efficacy: opportunities and challenges. *Nat. Rev. Genet.* 17 197–206. 10.1038/nrg.2016.12 26972588

[B88] Nour El Huda A. R. Norsidah K. Z. Nabil Fikri M. R. Hanisah M. N. Kartini A. Norlelawati A. T. (2018). DNA methylation of membrane-bound catechol-O-methyltransferase in Malaysian schizophrenia patients. *Psychiatry Clin. Neurosci.* 72 266–279. 10.1111/pcn.12622 29160620

[B89] Okada S. Morinobu S. Fuchikami M. Segawa M. Yokomaku K. Kataoka T. (2014). The potential of SLC6A4 gene methylation analysis for the diagnosis and treatment of major depression. *J. Psychiatr. Res.* 53 47–53. 10.1016/j.jpsychires.2014.02.002 24657235

[B90] Patkar A. A. Pae C. U. (2013). Atypical antipsychotic augmentation strategies in the context of guideline-based care for the treatment of major depressive disorder. *CNS Drugs* 27(Suppl. 1) S29–S37. 10.1007/s40263-012-0031-0 23709359

[B91] Paykel E. S. (1998). Remission and residual symptomatology in major depression. *Psychopathology* 31 5–14. 10.1159/000029018 9500681

[B92] Philibert R. A. Plume J. M. Gibbons F. X. Brody G. H. Beach S. R. (2012). The impact of recent alcohol use on genome wide DNA methylation signatures. *Front. Genet.* 3:54. 10.3389/fgene.2012.00054 22514556 PMC3322340

[B93] Pillinger T. McCutcheon R. A. Vano L. Mizuno Y. Arumuham A. Hindley G. (2020). Comparative effects of 18 antipsychotics on metabolic function in patients with schizophrenia, predictors of metabolic dysregulation, and association with psychopathology: a systematic review and network meta-analysis. *Lancet Psychiatry* 7 64–77. 10.1016/s2215-0366(19)30416-x 31860457 PMC7029416

[B94] Pinney S. E. (2014). Mammalian non-CpG methylation: stem cells and beyond. *Biology (Basel)* 3 739–751. 10.3390/biology3040739 25393317 PMC4280509

[B95] Poo S. X. Agius M. (2014). Atypical anti-psychotics in adult bipolar disorder: current evidence and updates in the NICE guidelines. *Psychiatr. Danub.* 26(Suppl. 1) 322–329. 25413559

[B96] Powell T. R. Smith R. G. Hackinger S. Schalkwyk L. C. Uher R. McGuffin P. (2013). DNA methylation in interleukin-11 predicts clinical response to antidepressants in GENDEP. *Transl. Psychiatry* 3:e300. 10.1038/tp.2013.73 24002086 PMC3784763

[B97] Pratt L. A. Brody D. J. Gu Q. (2017). Antidepressant use among persons aged 12 and over: United States, 2011-2014. *NCHS Data Brief* 283 1–8. 29155679

[B98] Rush A. J. Fava M. Wisniewski S. R. Lavori P. W. Trivedi M. H. Sackeim H. A. (2004). Sequenced treatment alternatives to relieve depression (STAR^∗^D): rationale and design. *Control Clin. Trials* 25 119–142. 10.1016/s0197-2456(03)00112-0 15061154

[B99] Rush A. J. Trivedi M. H. Wisniewski S. R. Nierenberg A. A. Stewart J. W. Warden D. (2006). Acute and longer-term outcomes in depressed outpatients requiring one or several treatment steps: a STAR^∗^D report. *Am. J. Psychiatry* 163 1905–1917. 10.1176/ajp.2006.163.11.1905 17074942

[B100] Ryan J. Pilkington L. Neuhaus K. Ritchie K. Ancelin M. L. Saffery R. (2017). Investigating the epigenetic profile of the inflammatory gene IL-6 in late-life depression. *BMC Psychiatry* 17:354. 10.1186/s12888-017-1515-8 29070016 PMC5657056

[B101] Rybakowski J. K. (2011). Lithium in neuropsychiatry: a 2010 update. *World J. Biol. Psychiatry* 12 340–348. 10.3109/15622975.2011.559274 21361856

[B102] Sabunciyan S. Aryee M. J. Irizarry R. A. Rongione M. Webster M. J. Kaufman W. E. (2012). Genome-wide DNA methylation scan in major depressive disorder. *PLoS One* 7:e34451. 10.1371/journal.pone.0034451 22511943 PMC3325245

[B103] Schroeder M. Hillemacher T. Bleich S. Frieling H. (2012). The epigenetic code in depression: implications for treatment. *Clin. Pharmacol. Ther.* 91 310–314. 10.1038/clpt.2011.282 22205200

[B104] Shenker N. S. Ueland P. M. Polidoro S. van Veldhoven K. Ricceri F. Brown R. (2013). DNA methylation as a long-term biomarker of exposure to tobacco smoke. *Epidemiology* 24 712–716. 10.1097/EDE.0b013e31829d5cb3 23867811

[B105] Shi Y. Li M. Song C. Xu Q. Huo R. Shen L. (2017). Combined study of genetic and epigenetic biomarker risperidone treatment efficacy in Chinese Han schizophrenia patients. *Transl. Psychiatry* 7:e1170. 10.1038/tp.2017.143 28696411 PMC5538123

[B106] Smith A. K. Kilaru V. Klengel T. Mercer K. B. Bradley B. Conneely K. N. (2015). DNA extracted from saliva for methylation studies of psychiatric traits: evidence tissue specificity and relatedness to brain. *Am. J. Med. Genet. B Neuropsychiatr. Genet.* 168b 36–44. 10.1002/ajmg.b.32278 25355443 PMC4610814

[B107] Song L. James S. R. Kazim L. Karpf A. R. (2005). Specific method for the determination of genomic DNA methylation by liquid chromatography-electrospray ionization tandem mass spectrometry. *Anal. Chem.* 77 504–510. 10.1021/ac0489420 15649046

[B108] Tadić A. Müller-Engling L. Schlicht K. F. Kotsiari A. Dreimüller N. Kleimann A. (2014). Methylation of the promoter of brain-derived neurotrophic factor exon IV and antidepressant response in major depression. *Mol. Psychiatry* 19 281–283. 10.1038/mp.2013.58 23670489

[B109] Takeuchi N. Nonen S. Kato M. Wakeno M. Takekita Y. Kinoshita T. (2017). Therapeutic response to paroxetine in major depressive disorder predicted by DNA methylation. *Neuropsychobiology* 75 81–88. 10.1159/000480512 29131015

[B110] Tang H. Dalton C. F. Srisawat U. Zhang Z. J. Reynolds G. P. (2014). Methylation at a transcription factor-binding site on the 5-HT1A receptor gene correlates with negative symptom treatment response in first episode schizophrenia. *Int. J. Neuropsychopharmacol.* 17 645–649. 10.1017/s1461145713001442 24331356

[B111] Uher R. Perroud N. Ng M. Y. Hauser J. Henigsberg N. Maier W. (2010). Genome-wide pharmacogenetics of antidepressant response in the GENDEP project. *Am. J. Psychiatry* 167 555–564. 10.1176/appi.ajp.2009.09070932 20360315

[B112] Venugopal D. Shivakumar V. Subbanna M. Kalmady S. V. Amaresha A. C. Agarwal S. M. (2018). Impact of antipsychotic treatment on methylation status of Interleukin-6 [IL-6] gene in Schizophrenia. *J. Psychiatr. Res.* 104 88–95. 10.1016/j.jpsychires.2018.07.002 30005373

[B113] Vialou V. Feng J. Robison A. J. Nestler E. J. (2013). Epigenetic mechanisms of depression and antidepressant action. *Annu. Rev. Pharmacol. Toxicol.* 53 59–87. 10.1146/annurev-pharmtox-010611-134540 23020296 PMC3711377

[B114] Wagner J. R. Busche S. Ge B. Kwan T. Pastinen T. Blanchette M. (2014). The relationship between DNA methylation, genetic and expression inter-individual variation in untransformed human fibroblasts. *Genome Biol.* 15:R37. 10.1186/gb-2014-15-2-r37 24555846 PMC4053980

[B115] Wagner S. Kayser S. Engelmann J. Schlicht K. F. Dreimüller N. Tüscher O. (2019). Plasma brain-derived neurotrophic factor (pBDNF) and executive dysfunctions in patients with major depressive disorder. *World J. Biol. Psychiatry* 20 519–530. 10.1080/15622975.2018.1425478 29334322

[B116] Walton E. Hass J. Liu J. Roffman J. L. Bernardoni F. Roessner V. (2016). Correspondence of DNA Methylation between blood and brain tissue and its application to schizophrenia research. *Schizophr. Bull.* 42 406–414. 10.1093/schbul/sbv074 26056378 PMC4753587

[B117] Wang K. Li M. Hakonarson H. (2010). Analysing biological pathways in genome-wide association studies. *Nat. Rev. Genet.* 11 843–854. 10.1038/nrg2884 21085203

[B118] Wang L. Xia Y. Chen Y. Dai R. Qiu W. Meng Q. (2019). Brain banks spur new frontiers in neuropsychiatric research and strategies for analysis and validation. *Genomics Proteomics Bioinformatics* 17 402–414. 10.1016/j.gpb.2019.02.002 31811942 PMC6943778

[B119] Wang P. Lv Q. Mao Y. Zhang C. Bao C. Sun H. (2018a). HTR1A/1B DNA methylation may predict escitalopram treatment response in depressed Chinese Han patients. *J. Affect. Disord.* 228 222–228. 10.1016/j.jad.2017.12.010 29275155

[B120] Wang P. Zhang C. Lv Q. Bao C. Sun H. Ma G. (2018b). Association of DNA methylation in BDNF with escitalopram treatment response in depressed Chinese Han patients. *Eur. J. Clin. Pharmacol.* 74 1011–1020. 10.1007/s00228-018-2463-z 29748862

[B121] Xia Y. Dai R. Wang K. Jiao C. Zhang C. Xu Y. (2019). Sex-differential DNA methylation and associated regulation networks in human brain implicated in the sex-biased risks of psychiatric disorders. *Mol. Psychiatry* 26 835–848. 10.1038/s41380-019-0416-2 30976086 PMC6788945

[B122] Yan J. Zierath J. R. Barrès R. (2011). Evidence for non-CpG methylation in mammals. *Exp. Cell Res.* 317 2555–2561. 10.1016/j.yexcr.2011.08.019 21925168

[B123] You C. Wu S. Zheng S. C. Zhu T. Jing H. Flagg K. (2020). A cell-type deconvolution meta-analysis of whole blood EWAS reveals lineage-specific smoking-associated DNA methylation changes. *Nat. Commun.* 11:4779. 10.1038/s41467-020-18618-y 32963246 PMC7508850

[B124] Yu H. Yan H. Wang L. Li J. Tan L. Deng W. (2018). Five novel loci associated with antipsychotic treatment response in patients with schizophrenia: a genome-wide association study. *Lancet Psychiatry* 5 327–338. 10.1016/s2215-0366(18)30049-x 29503163

[B125] Zhang H. S. Ke X. Y. Hu L. L. Wang J. Gao L. S. Xie J. (2018). Study on the epigenetic methylation modification of bipolar disorder major genes. *Eur. Rev. Med. Pharmacol. Sci.* 22 1421–1425. 10.26355/eurrev_201803_14489 29565503

